# Enhanced Antifungal Efficacy through Controlled Delivery of Amphotericin B Loaded in Polyetheramine-Epoxide Nanogels

**DOI:** 10.1021/acspolymersau.5c00037

**Published:** 2025-07-25

**Authors:** Julia S. Reinaldi, Heber E. Andrada, Ana F. A. P. Cunha, Bruno A. Fico, Felipe B. Alves, Renato P. Orenha, Renato L. T. Parreira, Regina H. Pires, Fabián Vaca Chávez, Carolina E. Tissera, O. Fernando Silva, Mariana A. Fernandez, Aline R. Passos, Eduardo F. Molina

**Affiliations:** † 92917 Universidade de Franca, Av. Dr. Armando Salles Oliveira 201, Franca, SP 14404-600, Brazil; ‡ 28217 Universidad Nacional de Córdoba, Ciudad Universitaria, X5000HUA Córdoba, Argentina; § Consejo Nacional de Investigaciones Científicas y Técnicas (CONICET), Instituto de Investigaciones en Fisicoquímica de Córdoba (INFIQC), X5000HUA Córdoba, Argentina; ∥ Brazilian Synchrotron Light Laboratory, Brazilian Centre for Research in Energy and Materials, 13083-1000 Campinas, Brazil

**Keywords:** click reaction, nanogel, spherical particles, ultra-small angle X-ray scattering, drug loading, sustainable release, antifungal

## Abstract

Polymeric nanomaterials have emerged as promising carriers for drug delivery systems, offering improved therapeutic efficacy and reduced toxicity. In this study, we present an environmentally friendly and scalable approach for engineering nanogels as an innovative delivery platform for Amphotericin B (AmB), which is a potent antifungal agent. The nanogel system, named NanoT, was synthesized via an amine–epoxide reaction, enabling effective encapsulation and sustained release of AmB. Comprehensive physicochemical characterization was conducted using transmission electron microscopy (TEM), dynamic light scattering (DLS), ζ potential analysis, proton nuclear magnetic resonance (1H-NMR), atomic force microscopy (AFM), Fourier transform infrared spectroscopy (FTIR), and synchrotron-based ultra-small angle X-ray scattering (USAXS). These analyses confirmed the successful formation of spherical nanogels and provided insights into their structural features. Additionally, molecular simulations indicated noncovalent interactions between AmB and the nanogel particles, supporting polymer-drug interactions. The NanoT system achieved an AmB loading capacity of approximately 55%. Notably, encapsulation promoted the formation of AmB superaggregates, which facilitated a controlled release of the active drug, leading to a 4-fold enhancement in antifungal activity. Mechanistic studies suggest that the antifungal efficacy of NanoT is attributed to both the sustained release of AmB and the electrostatic interactions with fungal cell surfaces. Overall, this study demonstrates the potential of amine–epoxide-based nanogels as effective carriers for antifungal therapeutics and contributes significantly to the development of advanced polymer-based drug delivery systems.

## Graphical abstract

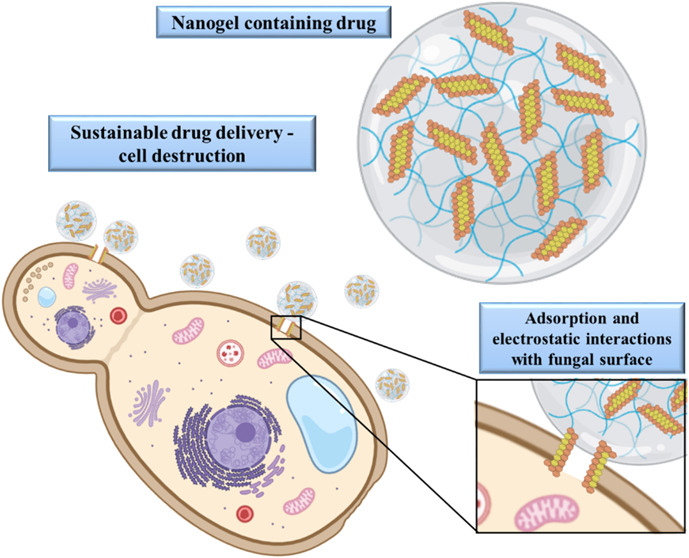

## 1 Introduction

Globally, fungal infections affect more than one billion people annually, posing a significant public health burden. Fungal infections vary in severity, ranging from mild superficial infections to life-threatening systemic diseases. Individuals with compromised immune systems whether due to underlying illnesses, aging, or immunosuppressive treatments such as corticosteroids are at a heightened risk of developing severe fungal infections.^1^ Therefore, addressing these challenges through the development of novel antifungal agents and advanced drug delivery systems is of critical importance for improving treatment efficacy and patient outcomes.

The use of macromolecular swollen networks (polymer-based particles known as gels) represents a promising strategy for combating fungal infections, particularly those associated with drug resistance.^2,3^ Their tunable composition enables the design of gel-based systems with specific physicochemical properties, allowing for optimized drug delivery and enhanced antifungal efficacy.^4^ Microgels and nanogels are soft and highly penetrable networks with an internal gel-like structure that swells in the presence of water or organic solvents. Their ability to respond to external stimuli, including pH, ionic strength, and temperature, makes them highly versatile carriers for various biomedical applications, particularly in drug delivery and healthcare.^5–9^ These gels offer several advantages like: (i) ability to retain their colloidal stability and swelling degree in an aqueous environment; (ii) the possibility to introduce functional groups at different positions; (iii) are formed by three-dimensional networks which are physically or chemically cross-linked; and (iv) fine-tuning the hydrophobic/hydrophilic balance of networks based on monomers used during the synthesis, beneficial for uptake-release purposes of active substances.^10,11^

Souza et al.^12^ evaluated the antimicrobial efficacy of amphotericin B (AmB)-loaded polymeric nanocarriers in combination with monoclonal antibodies (mAbs), demonstrating a significant enhancement in antifungal activity. The AmB was encapsulated within polylactic acid (PLA) and polycaprolactone (PCL) nanoparticles, which exhibited potent in vitro antifungal activity against a range of clinically relevant fungal pathogens, including *Candida albicans, Histoplasma capsulatum*, and *Cryptococcus neoformans*. Horvat et al.^13^ designed poly­(glycidol)-based nanogels for the encapsulation of the AmB, aiming to achieve sustained drug release. The study demonstrated that the antifungal efficacy of the nanogel-encapsulated AmB was significantly enhanced. This improved activity was attributed to the increased uptake of the nanogels by fungal cells, likely facilitated by the amphiphilic nature of the polymer components comprising the nanogels. Kim et al.^14^ developed a targeted drug delivery system based on lipopeptide-modified poly­(lactic-*co*-glycolic acid) (PLGA) micelles for the encapsulation of AmB, which exhibited enhanced antifungal efficacy in both in vitro and in vivo models. These functionalized micelles demonstrated selective antifungal activity against drug-resistant *Candida albicans*, highlighting their potential as promising therapeutic candidates for the treatment of resistant fungal infections.

In this study, nanogels were prepared by a simple and green click reaction (catalyst-free and water as unique solvent) based on the nucleophilic ring opening of a cyclic ether (epoxide monomer) with amines from a Jeffamine precursor. The literature has demonstrated the straightforward synthesis and biological application of amine-epoxide-based gels.^15^ The size and physicochemical properties of these gels can be finely tuned by varying the structural characteristics of the epoxide and polyetheramine components, enabling customization for several applications.^16–18^ The goal of the study was to evaluate, for the first time, this class of nanogels as an engineering carrier with optimal antifungal properties (containing AmB drug), which have a significant minimal inhibitory concentration (MIC) decrease with a sustainable profile release of AmB. The nanogel was in-depth characterized by transmission electron microscopy (TEM), dynamic light scattering (DLS) following the surface charge (ζ potential) of the particles, proton nuclear magnetic resonance ^1^H NMR measurements, atomic force microscopy (AFM), and Fourier transform infrared spectroscopy (FTIR). Moreover, the mechanism of nanogel formation and interaction between polymer and drug were evaluated by ultra-small angle X-ray scattering (USAXS) and computational studies, respectively. The in situ release profile of AmB drug loaded into nanogels was evaluated by UV–vis spectroscopy.

## 2 Materials and Methods

### 2.1 Materials

Poly­(ethylene glycol) diglycidyl ether (PEGDE, C_3_H_5_O_2_-(C_2_H_4_O)­n-C_3_H_5_O, Mw = 500 g mol^–1^; CAS 26403-72-5), Trimethylolpropane tris­[poly­(propylene glycol), amine-terminated] ether (Jeffamine T403, Mw = 440 g mol^–1^; CAS 39423-51-3), and amphotericin B (AmB, Mw = 924 g mol^–1^; CAS 1397-89-3) were purchased from Sigma-Aldrich. All reagents were used as received.

### 2.2 Synthesis of the Unloaded and Drug-Loaded Polyetheramine-Epoxide Gels

Polymeric gel network was synthesized by reacting the terminal amine groups of Jeffamine T403 with the cyclic ether from PEGDE using water as unique solvent, as reported elsewhere.^15,19^ The process followed a two-step approach, as illustrated in Scheme 1. In the first step, the monomers were dissolved in deionized water at a concentration of 150 mg mL^–1^ (15 wt %) in a molar 1:1 ratio. The resulting solution was incubated in a water bath at 65 °C for 15 min (without agitation) to initiate prepolymer formation. In the second step, the prepolymer solution was diluted to a concentration of 1.5 mg mL^–1^ (0.15 wt %) using deionized water and further incubated at 65 °C for 30 min to promote final colloidal particle formation (Scheme 1, second step). After the synthesis, the solution was neutralized and purified by membrane dialysis. The final polymeric nanogels were named here as *NanoT*.

**Scheme 1:**
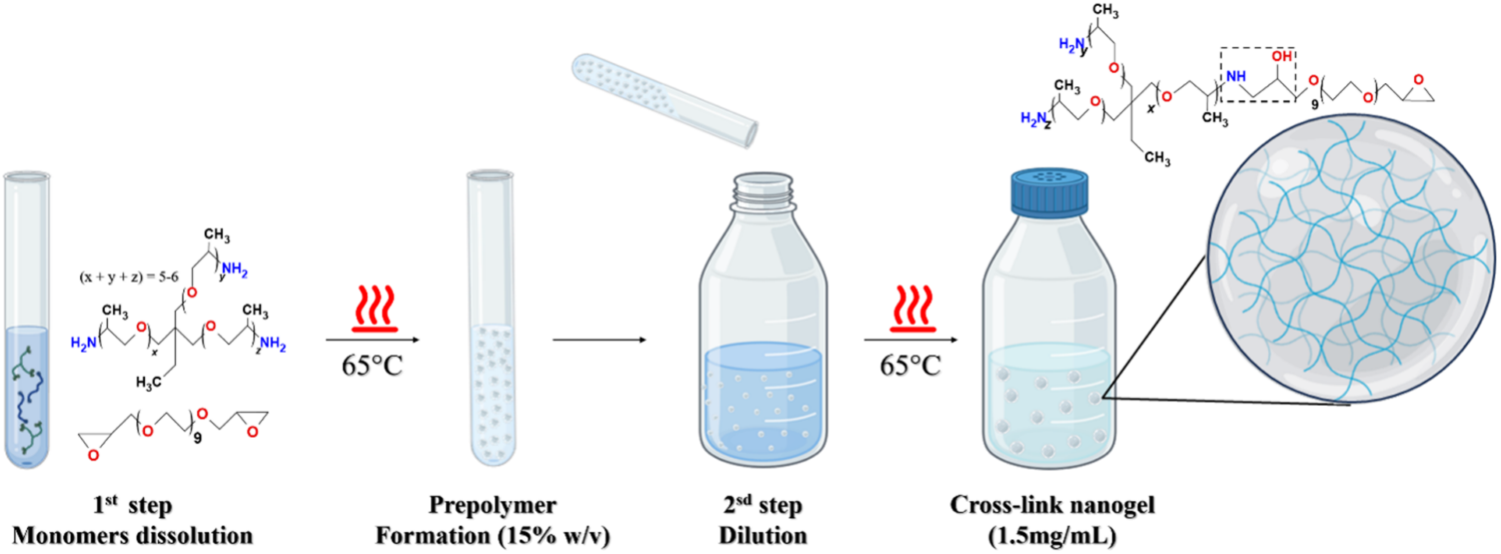
Representation of the NanoT Formation Process Based on Trimethylolpropane Tris [poly­(propylene Glycol), Amine-Terminated] Ether (Jeffamine T403) and Poly­(ethylene Glycol) Diglycidyl Ether (PEGDE) Diepoxy Poly­(ethylene glycol) (DPEG) Using Water as Solvent

To incorporate Amphotericin B (AmB) into the nanogel, freshly prepared NanoT was mixed with AmB to achieve final concentrations of 16 μg/mL of AmB for antimicrobial test and 64 μg/mL for the UV–vis spectrum (named here as *NanoT-AmB*). This relationship was chosen due to the detection of amphotericin B (UVvis spectrum) used in the work. The mixture was incubated for 24 h to allow drug encapsulation.

### 2.3 Instrumental Characterization

The hydrodynamic diameter (*D*_h_), polydispersity index (PDI), and zeta potential (ζ) of NanoT were evaluated using a ZSU3100 Zetasizer Lab Blue Malvern Instruments equipped with an OBIS solid state laser source (λ = 633 nm). Measurements of the *D*_h_ and ζ of NanoT were carried out at different pH (9, 7, and 5) to observe the colloidal particles’ response. In addition, experiments were conducted as a function of temperature, from 25 to 65 °C. The measurements were repeated three times (independently), and the dates were expressed as mean ± standard deviation (SD) (*n* = 3). The shape and size of the NanoT formulations were observed using Transmission Electron Microscopy (TEM) with a JEM 100CXII JEOL instrument operating at 100 kV. For the analysis, a drop of the nanogel solution was deposited onto a copper grid and allowed to dry at room temperature for 1h before measurement. Confirmation of the formation of the NanoT was performed using a PerkinElmer Frontier spectrometer equipped with an attenuated total reflection (ATR) accessory. The FTIR spectra were collected over the range of 4000–700 cm^–1^ by averaging 30 scans at a maximum resolution of 2 cm^–1^. ^1^H NMR measurements were performed on aqueous solutions of both the monomers (Jeffamine T403 and DPEG) and the NanoT synthesis reaction products obtained from their combination. The equipment used for the ^1^H NMR measurements was a Spinsolve 80 Ultra, manufactured by Magritek. This is one of the most powerful tools currently available for elucidating molecular structures, particularly organic molecules, in aqueous solution. Atomic force microscopy (AFM) was carried out using a Scanning Probe Microscope, Agilent Technologies model 5500, operating inside an acoustic and vibration isolation chamber in tapping mode. The measurement was performed using an AFM probe with an aluminum reflective coating on the back of the cantilever, with a force constant and resonance frequency of approximately 5 N/m and 170 kHz, respectively, and a tip radius of less than 10 nm (Tap150Al-G; BudgetSensors). A freshly cleaved 0.5 cm × 0.5 cm ultraflat silicon crystal was used as the substrate for the experiments. The NanoT solution was immobilized on the bare crystal surface through electrostatic interactions. A drop of nanogel dispersion with a polymer concentration of 0.15 wt % was deposited on the crystal surface and left to interact for 3 to 5 min. Excess sample was then removed by tilting the crystal onto a paper surface. Subsequently, the silicon crystal was transferred to a desiccator containing silica gel (at room temperature and atmospheric pressure) until the surface was visibly dry. Finally, the prepared samples were mounted on the microscope and measured in air.

### 2.4 In Situ Formation of Nanogels by Ultra-small Angle X-ray Scattering (USAXS)

*In situ* formation of NanoT particles was conducted using USAXS at the Cateretê beamline of the Brazilian National Synchrotron Light Laboratory (LNLS).^20^ For this purpose, the initial stage of the synthesis (1st step) was conducted following the methodology outlined in Scheme 1, resulting in the formation of a prepolymer at a concentration of 15 wt %. Subsequently, the prepolymer solution was diluted to 1 wt % to facilitate analysis using ultra-small angle X-ray Scattering (USAXS). Then, the prepolymer diluted solution was loaded into a quartz capillary with a diameter of 1.5 mm. The formation/mechanism of NanoT particles was monitored as a function of temperature, ranging from 25 to 60 °C with a ramp rate of 5 °C/min, using a Peltier-controlled heating system. A 40 × 40 μm X-ray beam with an energy of 9 keV was focused onto the sample. The scattered X-rays were detected by a Pimega 540D detector positioned 28 m downstream from the sample. The USAXS profiles were analyzed using an open-source scattering analysis software, SasView 6.0.1.^21^

### 2.5 Computational Study

The structure of the complex established between the Amphotericin B molecule and a representative polymeric structure of the nanogel (MEP_111_) was optimized (Amphotericin B^···.^MEP_111_) without applying geometric constraints (Figure 1), and vibrational frequency calculations were carried out using the BLYP functional,^22,23^ incorporating Grimme’s D3­(BJ) dispersion corrections with Becke–Johnson damping.^24^ The Def2–TZVP basis set was employed consistently across these calculations.^25^ To improve computational efficiency, the RIJCOSX approximation was applied,^26^ with Coulomb integrals managed by the RI–J^24^ method using the Def2/J auxiliary basis set.^27^ A vibrational frequency analysis confirmed that each optimized geometry represented a true energy minimum by showing no imaginary frequencies, supporting the accuracy of the computational model. These calculations were executed in the ORCA software package.^28^ To investigate the chemical bonding mechanism, the EDA–NOCV methodology was utilized.^29–31^ These calculations were conducted in the Amsterdam Density Functional (ADF) software;^32,33^ at the BLYP–D3­(BJ) level of theory with the TZ2P basis set.^34^ Scalar relativistic effects were incorporated self-consistently using the zero-order regular approximation (ZORA).^35^ The ZORA–BLYP–D3­(BJ)/TZ2P computational model effectively elucidated the bonding mechanisms in noncovalent interactions.^36,37^

**Figure 1:**
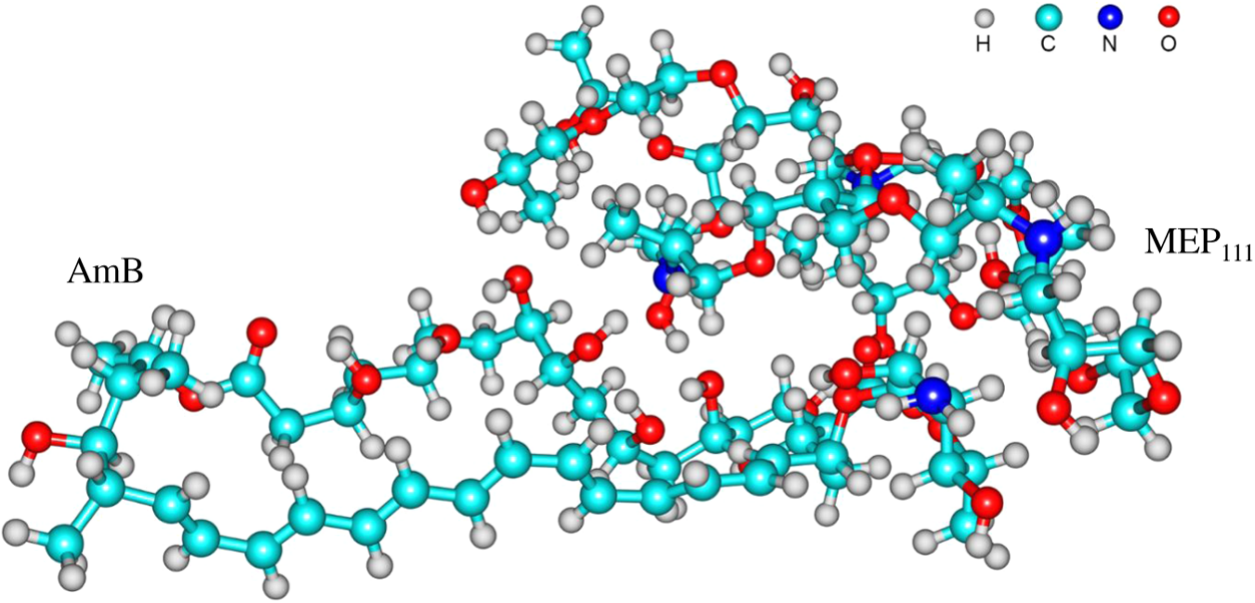
Optimized geometry of the Amphotericin B (left)^···^MEP_111_(right) complex.

### 2.6 Drug Encapsulation Efficiency (EE%)

The encapsulation efficiency coefficient (EE%) of a drug is a measure that indicates how effectively the drug is incorporated or retained within a delivery system, in this case, nanogels. This measurement is used to evaluate how well the drug has been integrated into the system and, therefore, how much of the drug is available for release. To determine this, a nanogel solution containing 0.6 g of polymer was prepared, to which 320 μg of AmB was added. The solution was then centrifuged at 7500 rpm for 30 min using Amicon Ultra 15 mL centrifugal filter units (Sigma-Aldrich) with a molecular weight cutoff (MWCO) of 100 kDa. Following centrifugation, the filtrate was collected, and its absorbance was measured by UV–visible spectroscopy. The absorption band at 408 nm was used to monitor the release of amphotericin B (AmB) in water, as it corresponds to the π–π* electronic transition of the polyene chromophore in the monomeric form of the drug. In aqueous media, AmB tends to remain monomeric under dilute conditions, and this form shows a strong and specific absorption peak at 408 nm.^38–45^ This wavelength is widely used for quantitative analysis due to its linear response and minimal interference from aggregates or formulation components. Based on the calculations performed using the absorbance at 408 nm, the encapsulation efficiency of AmB in the nanogels was determined (eq 1, see details of the encapsulation efficiency of AmB in the Supporting Information).

1 $${\mathrm{EE}\left(\%\right)}_{408\;\mathrm{nm}}=\frac{{\mathrm{mass}}_{\mathrm{AmB}\;\mathrm{Total}}-{\mathrm{mass}}_{\mathrm{AmB}\;\mathrm{free}}}{{\mathrm{mass}}_{\mathrm{AmB}\;\mathrm{Total}}}\times 100$$

### 2.7 In Vitro Drug Release Assays (UV–Vis)

Temporal release of loaded AmB into NanoT was evaluated by using NanoT-AmB formulation (188:1 μg/μg for NanoT:AmB: ratio, as described above). The release assays were carried out by using 0.6 g of Polymer +320 μg of AmB mixture which we refer to as NanoT-AmB, dispersed in 5 mL of PBS placed in a dialysis membrane bag, which was then placed in 100 mL of a PBS solution at controlled temperature 37 °C. The AmB molecules released were monitored by UV–Vis spectroscopy, using a Cary60 dual-beam spectrophotometer (Agilent Technologies) connected to an immersion probe (optical path length of 2 mm). The linearity of an analytical procedure refers to its ability to demonstrate that the measured absorbance is directly proportional to the analyte concentration in the sample. Quantitative determination of the cumulative AmB released was performed using a calibration curve constructed using the maximum absorbance values at λ_max_ = 408 nm of drug solutions at different concentrations from 0.5 to 64 mg L^–1^ (Figure S1). All the in vitro release experiments were performed with three independent samples, and the results are presented as the data average.

### 2.8 Minimum Inhibitory Concentration (MIC) and Minimum Fungicidal Concentration (MFC)

The MIC and MFC of amphotericin B (AmB, Sigma-Aldrich Co., St. Louis, MO) and AmB-incorporated nanogel against *Candida albicans* ATCC 90028 and *Candida glabrata* ATCC 2001 were determined according to the M27-S4 guidelines of the Clinical and Laboratory Standards Institute (CLSI, 2012). The inoculum was prepared in Roswell Park Memorial Institute (RPMI) 1640 medium, supplemented with 0.2% (w/v) glucose, and buffered with MOPS [3-(N-morpholino) propanesulfonic acid] at 0.165 mol/L, pH 7.0, to a final concentration of 2.5 × 10^3^ cells/mL. The concentration range tested was 0.004–2.0 μg/mL for free AmB and 0.37–187.5 μg/mL for AmB incorporated into the nanogel. Control wells with only culture medium, nanogel, and yeast without the drug were also included. *Candida krusei* ATCC 6258 and *C. parapsilosis* ATCC 22019 strains served as quality controls. After 48 h of incubation at 37 °C, MIC was determined using the fluorometric indicator resazurin at 0.01%, defined as the lowest product concentration that maintained a blue hue. MFC was determined by transferring 100 μL aliquots of each treated sample onto SDA plates and incubating at 37 °C for 48 h. The lowest product concentration that eliminated 99.9% of the initial inoculum on agar plates was recorded as the MFC (CLSI, 2012).^46^ The assays were performed in biological triplicate of the samples.

### 2.9 Hemolytic Activity Assay

Hemolytic activity was evaluated following the protocol described by Carpenter and Hoek.^47^ A 3% suspension of defibrinated sheep red blood cells (RBCs), obtained from a commercial kit (Laborclin Produtos para Laboratórios Ltd.), was prepared in phosphate-buffered saline PBS (pH 7.0). In Eppendorf tubes, 300 μL of the RBC suspension was mixed with 300 μL of NanoT at final concentrations of ∼5.8, 11.5, 23, and 46 μg/mL. The mixtures were incubated at room temperature for 3 h. Following incubation, the samples were centrifuged at 3000 rpm for 5 min. Subsequently, 100 μL of the supernatant from each sample was transferred to a 96-well microplate, and the absorbance was measured at 550 nm using a Libra S12 spectrophotometer. Distilled water and PBS (pH 7.0) were used as positive (PC) and negative (NC) controls, respectively. All assays were conducted in triplicate, and the results are presented as mean values.

## 3 Results and Discussion

### 3.1 Polyetheramine-Epoxide NanoT—Characterization and Mechanism of Formation

Figure 2 shows dynamic light scattering (DLS) measurement, transmission electron microscopy (TEM) images, and respective histogram of the NanoT particles. The prepared NanoT in water exhibits a Gaussian-type size distribution based on intensity, centered at 270 nm, alongside a number-based size distribution centered at 169 nm (Figure 2a). The latter is more closely aligned with the dimensions observed via transmission electron microscopy (TEM), as illustrated in Figure 2b, revealing both individual smaller particles and aggregates, predominantly dimers, with sizes that correlate with the peak in the number distribution indicated by dynamic light scattering (DLS). The difference in size measurements by TEM and DLS can be attributed to DLS not distinguishing between particle aggregates and individual particles. Consequently, the system comprises a variety of individual nanoscale particles as well as aggregates that may exist as dimers or larger clusters in solution, which is reflected in the DLS findings. A depth evaluation of different NanoT regions by TEM images also showed a well-defined spherical particles formed after the synthesis of amine-epoxide in water, with size varying from 60 to 80 nm (Figure 2c). Based on the statistical analysis of a population of 70 particles (occurrence frequency as a function of particle size), it can be observed that the average value of the particles is 65 ± 2 nm. It is noteworthy to observe the presence of particles with sizes ranging from 30 to 120 nm (Figure 2d). This variation can be explained by the fact that, during synthesis, the nanogels are formed from monomers cross-linking click reaction. Not all nanogels reach the same size in solution as each is produced from a different number of prepolymer particles. Consequently, the NanoT formulation exhibits a size distribution and a degree of polydispersity (PDI of ∼0.20) in solution.

**Figure 2:**
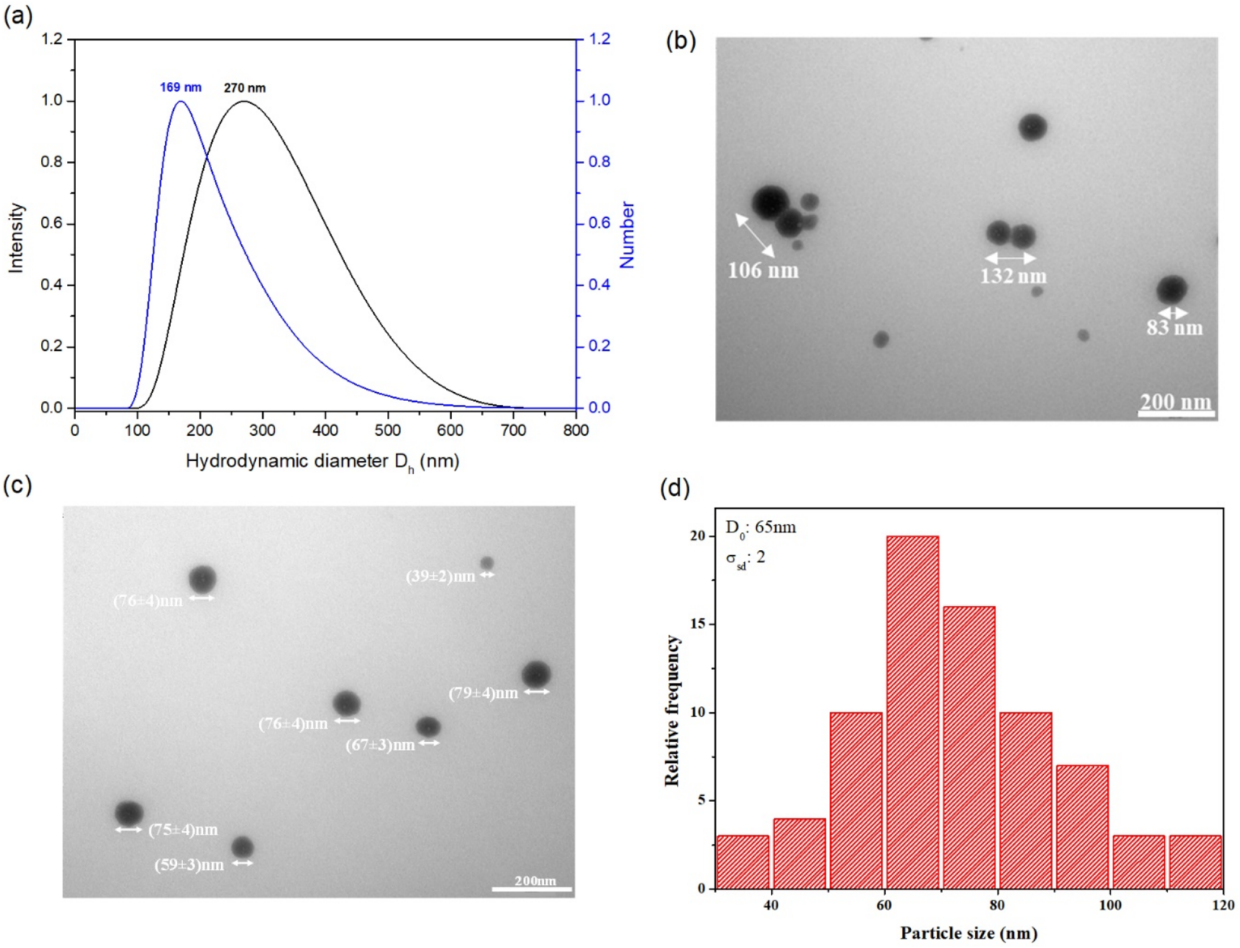
Size distribution (hydrodynamic diameter, *D*_h_) of the NanoT based on intensity (black line) and number size distribution (blue line); (b, c) representative of TEM images of NanoT; and (d) relative frequency versus particle size of NanoT system. The scale bar observed in the TEM images corresponds to 200 nm.

The dimensions and morphology of NanoT nanogels were further characterized by tapping-mode AFM. Atomic force microscopy (AFM) images (Figure 3a) indicated that the NanoT nanogels exhibited uniform and spherical morphology, which agree well with TEM results. The number-average particle height was 44 ± 2 nm, and the diameter was about 130 ± 10 nm (Figure 3b,c). Comparing the data obtained from DLS and TEM, it is evident that the size of the nanogels is like that obtained by AFM, although it appears to be slightly smaller. This is reasonable considering that the images were captured in air after water evaporation, and that, when deposited on the silicon substrate, the nanogels could deform due to interactions with the surface. It is important to emphasize that, while some particle aggregation may occur in solution, the nanogels exhibit a significantly positive surface charge due to the protonation of amine groups. This positive charge induces electrostatic repulsion between particles, effectively preventing their high aggregation.^15^ Thus, AFM results (Figure 3a–c), as well as TEM image (Figure 2c), show well-defined and uniform spherical particles. Figure 3d–f shows the ^1^H NMR spectrum obtained from solution of Jeffamine T-403, PEGDE, and NanoT nanogel.

**Figure 3:**
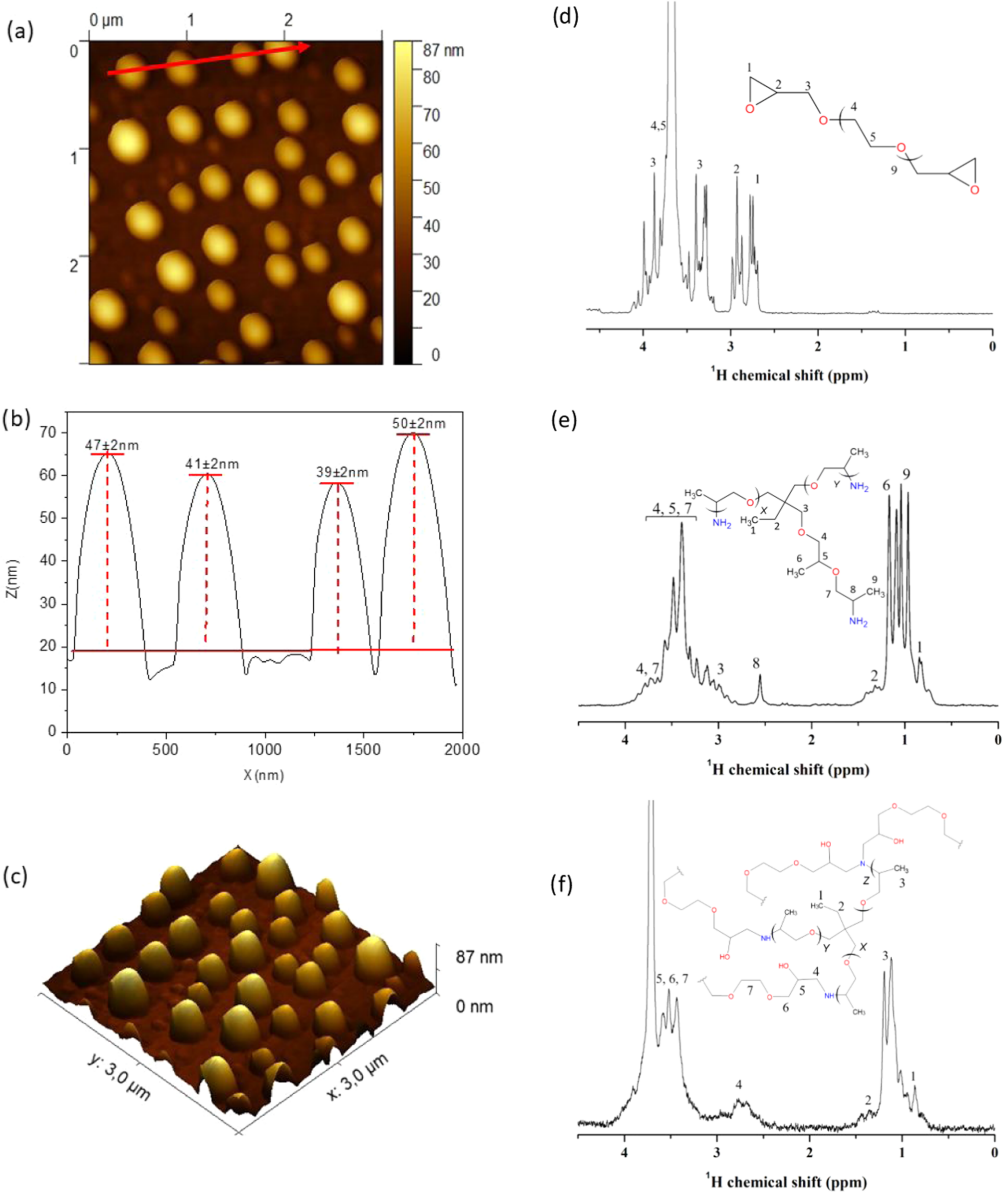
(a) Tapping-mode AFM image of NanoT in air, (b) height profile for the red line in (a), and (c) 3D structure of NanoT nanogels; ^1^H NMR spectrum of (d, e) PEGDE and Jeffamine T-403 monomers and (f) NanoT polymeric gel.

Figure 3d presents the ^1^H NMR spectrum of PEGDE in ultrapure water. The spectrum displays resonance signals at chemical shifts ranging from δ = 2.6 to 4.2 ppm, which are attributed to protons associated with the epoxide ring (−CH_2_–O), the tertiary carbon centers (−CH−) within the epoxide structure, the ether functional groups (−CH_2_–O−), and the methylene (−CH_2_−) units along the epoxide backbone. From Figure 3e, the polyetheramine-Jeffamine T-403 molecule is presented, with each peak identified with its respective protons. The spectrum shows a group of signals or peaks with low chemical shifts, between δ = 0.7 and 1.5 ppm, related to the protons of the (CH_3_-) and (−CH_2_−) groups in the ethyl branch as well as methyl groups near the terminal amine groups of Jeffamine. On the other hand, a grouping of peaks with higher shifts, between δ= 2.5 and 4 ppm, is observed, associated with protons from the ether – (CH_2_–O−), tertiary carbons (−CH−) near methyl groups, and primary amines (−CH-NH_2_). These peaks were previously observed by Tang et al.,^19^ who measured the ^1^H spectrum of Jeffamine T-403 in deuterated water. During the synthesis, NanoT nanogels are formed by monomer reaction in aqueous solution, resulting in peaks corresponding to both Jeffamine T-403 and the epoxide (see Figure 3f). It is important to note that during the reaction, the amine group from Jeffamine binds to the secondary carbon of the epoxide ring, causing ring opening and the formation of −OH group. The protons attached to the carbon where the amine group bonded appear around δ= 2.8 ppm, while the proton on the tertiary carbon that was part of the epoxide ring now appears around δ= 3.7 ppm. The peaks correspond to the signal of the protons involved in the amine-epoxide click reaction.

The obtained nanoT was characterized by Fourier transform infrared (FTIR) spectroscopy. Figure S2 shows the FTIR spectrum of monomers Jeffamine T403, PEGDE, and obtained NanoT. The typical bands of the epoxide rings (C–O) are observed around 850–950 cm^–1^, in addition to the bands corresponding to the symmetric and asymmetric stretching of the ether bonds (C–O–C) around 1100–1250 cm^–1^, respectively, and the band of the C–H bonds around 2860 cm^–1^ (Figure S2, black line). The FTIR spectrum of Jeffamine T403 (Figure S2, red line) present the main absorption bands about 1455 cm^–1^ and 1371 cm^–1^ attributed to the CH_2_/CH_3_ streching vibrations, the most intense banda around 1097 cm^–1^ attributed to the C–O streching vibration and a broad band at 1590 cm^–1^, which is assigned to the asymmetric streching vibration/deformation of the NH_2_ groups attached to the end of polyether backbone.^48^ After the amine-epoxide reaction (Figure S2, blue line) the band produced by the asymmetric stretching vibration of the NH_2_ group strong upshift to 1645 cm^–1^, following by the change of the band centered at 910 cm^–1^, characteristic of epoxide groups, probing the successful reaction by opening of the epoxide rings as they react with the amine-terminated groups from Jeffamine T403 to form the polymeric network. Additionally, a broad band around 3150–3700 cm^–1^ is noted, which is attributed to the formation of new hydroxyl groups (−OH).^49^

The structure evolution during the synthesis of NanoT particles was investigated *in situ* using USAXS. Figure 4a presents temperature-selected SAXS spectra from through the heating stage 45 < *t* < 60 °C. The polymeric gel particle formation exhibits temperature dependence. No structural features were observed in the USAXS curves between 25 and 40 °C, suggesting that the initial small structures (i.e., oligomeric species) were below the detection limit of the q-range probed by USAXS (Figure S3). The scattering curve at 45 °C shows a Guinier region at low *q* value (a clear plateau) which is followed by a power-law decay consistent with Porod’s law (I­(q) ∼ q^–4^). With increasing temperature, the Guinier plateau shifts toward low *q* values as the reaction time advances (Figure 4a), indicating that the individual nanoparticles increase in size is consistent with growth of the gyration radius (*R*_g_). The scattering data were fitted using the Guinier-Porod model, which accounts for the low-q Guinier regime and the high-q power-law decay characteristic of Porod behavior^50^ (see details in Supporting Information Table S1). The increase in Guinier radius (*R*_g_) with temperature (>45 °C) indicates a corresponding growth in the size (*R*_g_) of the NanoT particles (Figure 4b). The observation of a Porod exponent of −4 during the entire synthesis indicates that the NanoT particles developed smooth interfaces from the initial stages of their formation. The absence of a well-defined form factor is likely a result of significant polydispersity, as observed by electron microscopy analysis. A similar behavior has also been reported for polymeric nanogels.^51^ For the first time, USAXS has revealed the mechanism of amine-epoxide gel formation, after dilution to form intermediate polymer (prepolymer), demonstrating a growth size step—aggregation of particles above 45 °C, which drives the system’s evolution toward the formation of spherical polymeric particles. Figure 4c illustrates this mechanism that agrees with the literature.^19^

**Figure 4:**
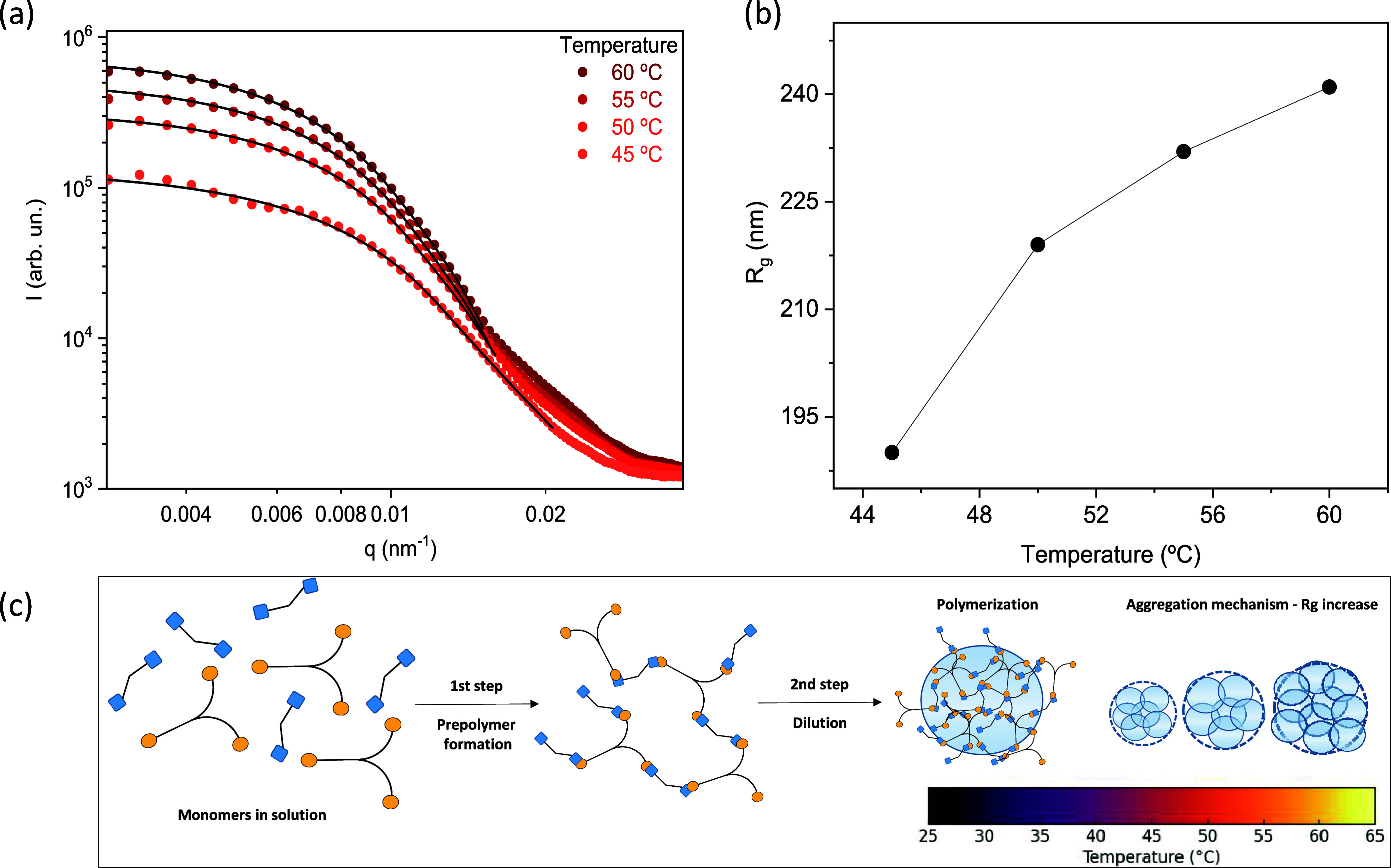
Temporal evolution of the (a) USAXS profile and (b) gyration radius (*R*_g_) during the synthesis of NanoT particles as a function of temperature and (c) illustration of possible NanoT polymerization-aggregation mechanism as a function of temperature.

### 3.2 Computational Studies

In addition to the physicochemical characterization of the nanogels, a computational study of the NanoT component MEP_111_ with Amphotericin B was carried out to gain insights into the drug-nanogel interactions. A simplified model of the NanoT network (MEP_111_) was assumed to facilitate the calculations, which may not fully represent the ionization state of the selected −NHR groups. The simplified model is likely to contribute valuable information to supplement the experimental drug delivery results presented below. An attraction effect between Amphotericin and B^···.^MEP_111_ was observed after optimization. The Energy Decomposition Analysis (EDA) method was employed to clarify the bonding mechanism between these two structures. The interaction energy, Δ*E*_int_,^27^ is composed of four main components

2 $$\Delta E_{\mathrm{int}}=\Delta V_{\mathrm{elstat}}+\Delta E_{\mathrm{Pauli}}+\Delta E_{\mathrm{oi}}+\Delta E_{\mathrm{disp}}$$

The electrostatic energy, Δ*V*_elstat_, represents the classical electrostatic interactions between the unperturbed charge distributions of the interacting fragments. The Pauli repulsion term, Δ*E*_Pauli_, captures destabilizing interactions between occupied orbitals, typically linked to steric effects. The orbital interaction energy, Δ*E*_oi_, accounts for charge transfer (interactions between occupied orbitals on one fragment and unoccupied orbitals on the other) and polarization (mixing of occupied and unoccupied orbitals due to the influence of the other fragment). The Δ*E*_disp_ component incorporates dispersion corrections, following the method proposed by Grimme et al.^25,52^

The bond between Amphotericin B^···.^MEP_111_ shows an attractive interaction energy (Δ*E*_int_ = −71.09 kcal mol^–1^). It occurs because the sum of all attractive energy terms: (i) Δ*V*_elstat_ (−91.54 kcal mol^–1^); (ii) Δ*E*_oi_ (−52.37 kcal mol^–1^); and (iii) Δ*E*_disp_ (−52.15 kcal mol^–1^), overcomes the Pauli repulsive energy Δ*E*_Pauli_ (124.97 kcal mol^–1^). Further, the larger weight of Δ*V*_elstat_ (47%) compared to Δ*E*_oi_ (27%) and Δ*E*_disp_ (27%) energetic components to Δ*V*_elstat_ + Δ*E*_oi_ + Δ*E*_disp_ indicates that the Amphotericin B^···.^MEP_111_ interaction has a predominantly noncovalent nature. The Natural Orbitals for Chemical Valence (NOCV) method provides insights into critical orbital interactions between Amphotericin and B^···.^MEP_111_ by decomposing the interaction into pairwise contributions from the most relevant molecular orbitals. Each orbital interaction within a particular chemical bond can be visualized through deformation density channels, Δρ_k_(r), where red areas represent electron density outflow and blue areas represent inflow. The NOCV method also quantifies the energetic contribution (Δ*E*_oi,k_) of each deformation density channel (Δρ_k_) to the overall orbital interaction energy (Δ*E*_oi_).^28,29^ The NOCV methodology shows Amphotericin B^···.^MEP_111_ interaction with relevant density deformation channel, Δρ_1_ (Figure Y), which is related to the σ-HN (MEP_111_)^···.^OH (Amphotericin B) chemical interaction (Figure 5). These interactions clearly revealed the effect of AmB on the final charge of the system (i.e., AmB-loaded NanoT), leading to a decrease of ζ-potential value from +26 mV (unloaded NanoT) to +5 mV (loaded NanoT), following the *D*_h_ augment from about 169 to 270 nm, respectively (see details in Table S2).

**Figure 5:**
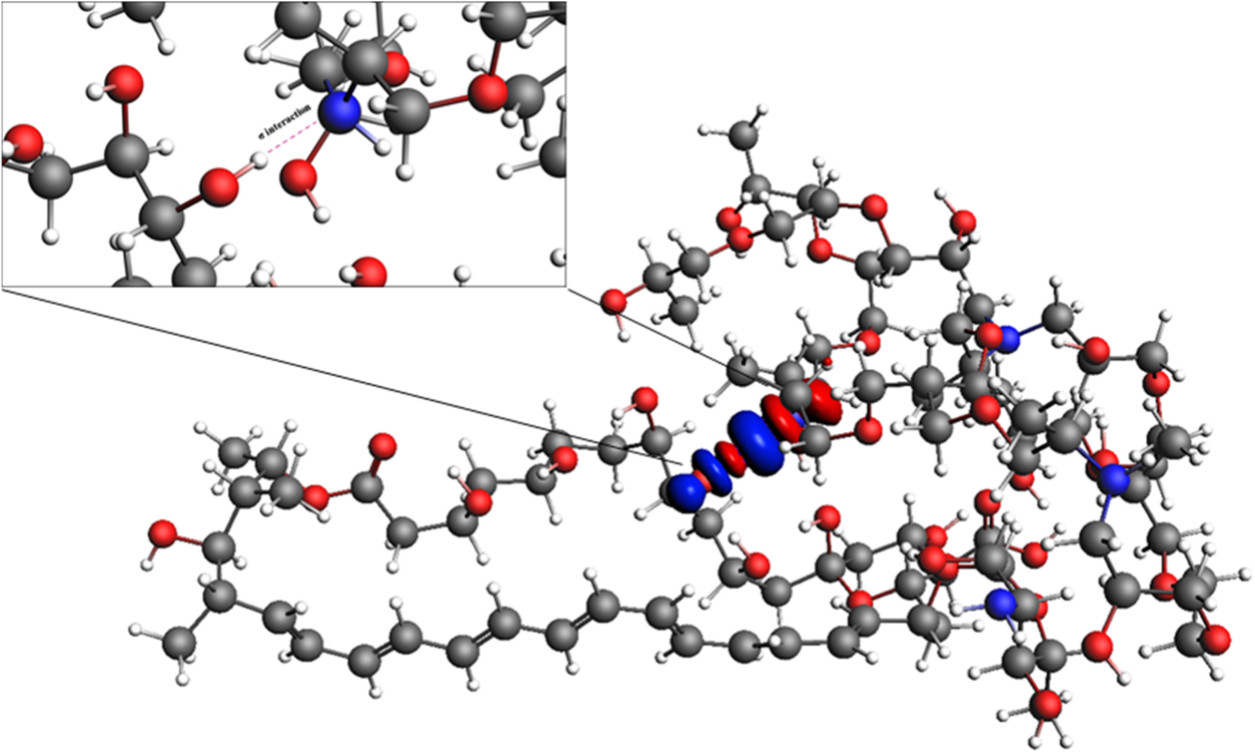
Surface plot of the first density deformation channel, Δρ_1_, with an isovalue of 0.001 au. Red regions denote electron density outflow, while blue regions indicate electron density inflow in the studied complex.

### 3.3 Amphotericin B (AmB) Drug Release: *In Situ* UV–Vis Studies

The amount of AmB drug encapsulated (EE%) in the NanoT system using eq 1 was about 53%. The UV–visible spectroscopy technique is particularly useful for characterizing AmB formulations since it has been shown to be very sensitive to the aggregation state of AmB and to interactions of the drug with other molecules.^53^ Figure 6a presents the UV–vis absorption spectra of AmB drug, NanoT, and the loaded NanoT-AmB. The UV–vis spectrum of free AmB (Figure 6a red line) displays absorption bands at approximately 408 nm, indicative of its monomeric form in aqueous solution, and at 339 nm, characteristic of its aggregated state (dimers or oligomers in solution),^54^ and no signal is observed by NanoT gel (Figure 6a, black line). Upon incorporation of AmB into the NanoT, notable modifications in UV–vis bands were observed (Figure 6a, blue line). The nanogel containing drug molecules induced a shift in both the intensity and position of the maximum absorption band, from 339 nm (AmB solution) to 325 nm (NanoT-AmB system). This feature in NanoT-AmB UV–vis spectra is due to the formation of superaggregates of AmB molecules (i.e., a hypsochromic shift in the λmax at 339 nm). The hypsochromic shift strongly suggests the interaction between the polymer network and AmB molecules, which are consistent with computational simulations, which indicate a noncovalent interaction between AmB and the polymeric network of the nanogel. The literature has shown this hypsochromic effect after obtention of AmB-lipid polymer hybrid nanoparticles, suggesting that the polymer-drug interactions provide advantages, leading AmB release with increasing its efficacy.^55^ Reports on Amphotericin B (AmB) superaggregates have highlighted several advantages, including reduced drug toxicity in both in vitro and in vivo models,^56,57^ as well as enhanced efficacy in the treatment of *Candida albicans* infections in murine models.^58^ These benefits are attributed to the dense structural organization of the superaggregates, which limits the accessibility of the polyene and carboxylic acid moieties, thereby reducing AmB’s affinity for cholesterol. Despite this aggregation, AmB retains high selectivity for ergosterol, which contributes to its reduced toxicity in human cells while maintaining potent antifungal activity.^59^ The cumulative release profile from AmB-loaded NanoT is shown in Figure 6b. The release rate of AmB achieved 15% during the initial 3 h (180 min). The initial release rate was higher compared to the gradual evolution of the drug released as a function of time. After 180 min, a gradual and slow release of AmB was obtained, reaching about 43% of the drug released after 48 h of assay. The release rate of AmB may be described by direct solvation (diffusion-controlled) of the drug molecules present on the surface (physisorbed) of the nanogels (initial step, 180 min), followed by the AmB amount hosted through the aggregate particles, leading to a gradual cumulative release of the AmB until ∼43%. The release mechanism of the Amb-loaded NanoT was evaluated by using mathematical models (see details in Table S3 and Figure S4) that have been developed to describe drug release.^60,61^ Figure S4 shows the release curves plotted by using different mathematical models. The curve of AmB-loaded NanoT was well fitted with the zero-order kinetic model (correlation coefficient *R* ^2^ of 0.993), where the drug-dissolved versus time was linear after 180 min of assays (see Figure S4a). The release profile of AmB-loaded into the nanogels suggests that the amine-epoxide polymeric network may serve effectively as a drug delivery system for sustained pharmacological action, given its consistent, time-dependent drug release rate. According to the Korsmeyer-Peppaśs equation (Table S3 and Figure 6c), a correlation coefficient *R* ^2^ of ∼0.979 and an *n* value of ∼0.41 for AmB-loaded NanoT are in line with the fact that the release of drug is governed by a Fickian diffusion. The controlled release of AmB is essential for maximizing its therapeutic efficacy, particularly in light of its physicochemical limitations such as poor aqueous solubility. In this context, unlike approved commercial lipid-based formulations such as AmBisome, the NanoT system is based on a nonlipidic platform capable of providing sustained release of AmB. By avoiding the use of lipid excipients, the NanoT formulation not only reduces production costs but also represents a more accessible and scalable therapeutic option.

**Figure 6:**
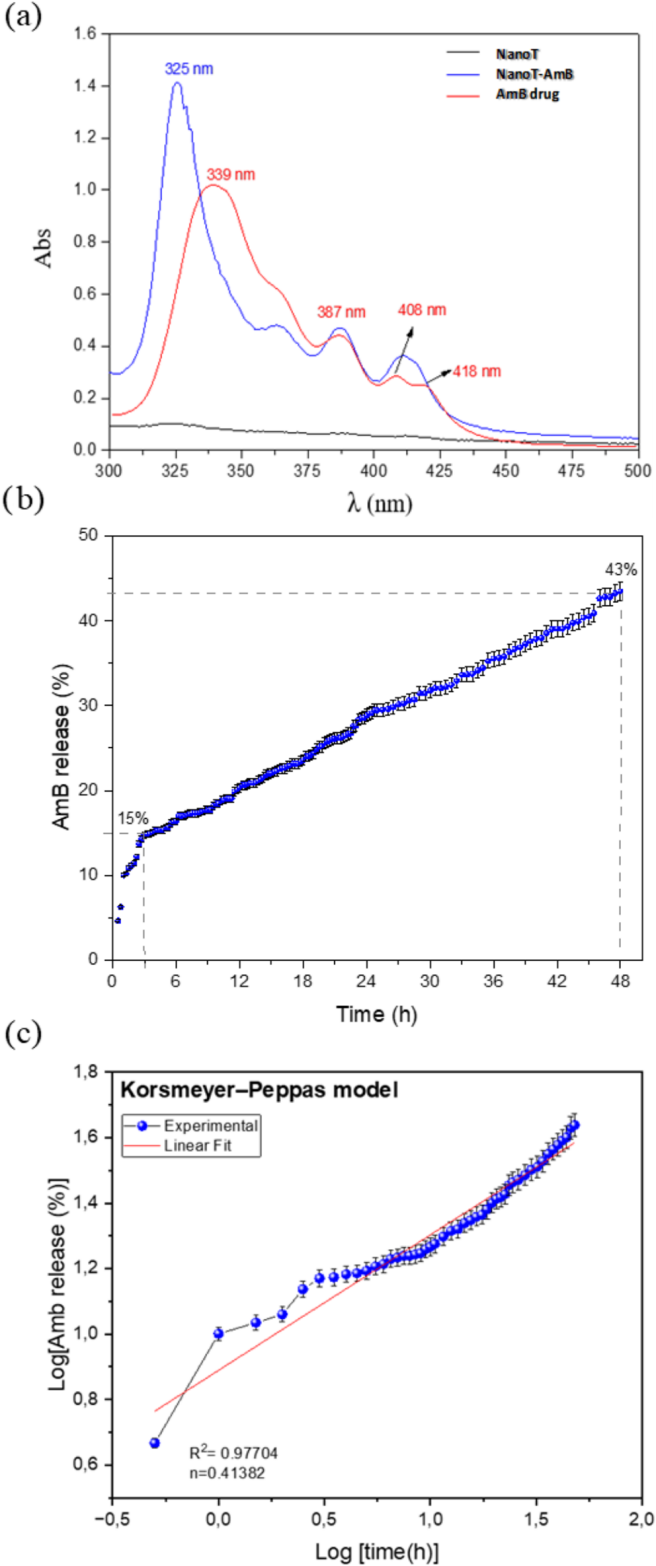
(a) UV–vis absorption spectra of amphotericin B (AmB), NanoT, and loaded NanoT-AmB. (b) Cumulative release of AmB from NanoT in PBS (pH 7.4). (c) Plot of Log­[AmB amount release (%)] against Log [time (h)].

### 3.4 Antifungal Mechanism of Loaded Amphotericin B Amine-Epoxide Nanogels

Figure 7 illustrates the antifungal activity of the unloaded NanoT formulation, AmB-loaded NanoT, and free AmB across varying concentrations. The minimum fungicidal concentration (MFC), determined by total viable colony counts (CFU/mL) shown in Figure 7a,b, indicated MFC values of 0.5 μg/mL for both *C. albicans* and *C. glabrata* when treated with free AmB. The AmB-loaded NanoT formulation promoted a 4-fold reduction in MFC, lowering it to 0.125 μg/mL for both species. The Minimum Inhibitory Concentration (MIC), determined based on resazurin color changes, revealed that free AmB exhibited MIC values of 0.5 μg/mL for *C. albicans* and 0.25 μg/mL for *C. glabrata* (Figure 7d,e, red wells). In contrast, the AmB-loaded NanoT formulation further reduced the MIC values to 0.125 μg/mL for *C. albicans* and 0.031 μg/mL for *C. glabrata* (Figure 7d,e, pink wells). Additionally, the quality control strains exhibited free AMB MIC values within the CLSI-recommended ranges, with MICs of 1 μg/mL for *C. krusei* ATCC 6258 and 0,5 μg/mL for *C. parapsilosis* ATCC 22019, respectively.

**Figure 7:**
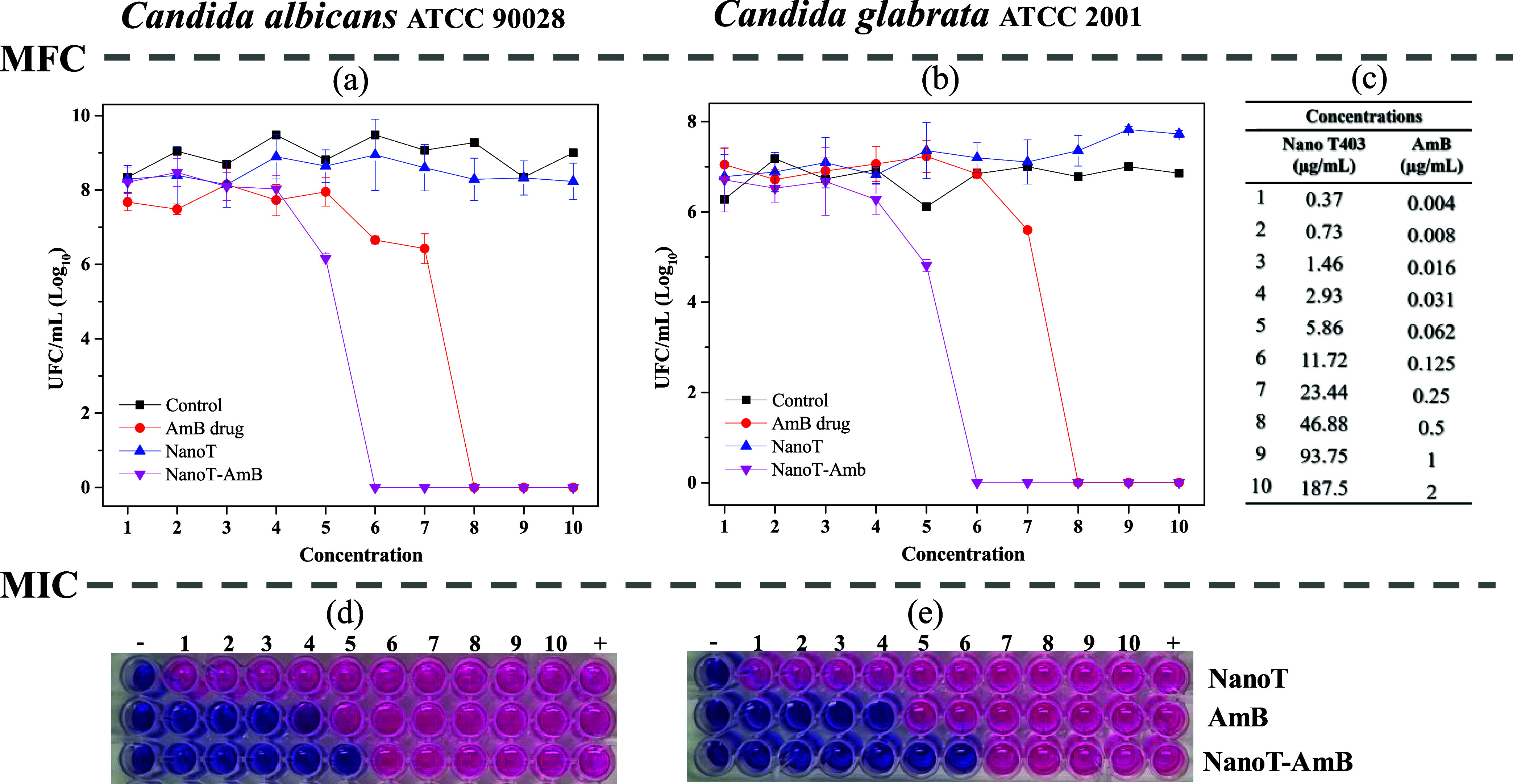
Antifungal activity of pure NanoT, AmB drug, and NanoT-AmB formulation against *Candida* species. Results for *C. albicans* (left column: a, d) and *C. glabrata* (right column: b, e) are shown. Minimum fungicidal concentration (MFC) was determined based on total viable counts (CFU/mL), as presented in (a) and (b). Concentrations of NanoT, AmB drug, and NanoT-AmB used in the MFC assays are shown in (c). Minimum inhibitory concentration (MIC) was assessed based on resazurin color changes in the microtiter plate wells, with blue indicating nonviable cells and pink indicating viable cells (d, e). Concentrations in the MIC assays followed a descending order, as indicated in (c). All tests were performed in triplicate.

The enhanced antifungal activity exhibited by NanoT-AmB may result from the following mechanisms: (i) the positive surface charge of the NanoT system, which promotes electrostatic interactions with the electronegative fungal surface, facilitating closer contact with the fungi; (ii) these NanoT–fungal surface interactions enhance the adsorption of the nanogel onto the fungal cell surface, promoting the translocation (release) of AmB to the ergosterol-rich cell membrane of the yeast, ultimately leading to cell death via AmB’s mechanism of action.

In general, antimicrobial polymers contain two key functional components: a cationic moiety and a hydrophobic moiety.^62^ The cationic group facilitates electrostatic interaction and adsorption to the negatively charged microbial membrane, while the hydrophobic group enables insertion into the lipid bilayer. This dual interaction typically results in membrane destabilization and could lead to cell lysis.^63,64^ It is important to note that the unloaded cationic NanoT formulation did not exhibit antifungal activity. This outcome may be attributed to the complex composition of the fungal cell wall, which is organized into distinct structural groups as well as the lack of sufficient hydrophobic groups in the NanoT system capable of effectively penetrating the fungal membrane and causing damage. However, a key aspect of the proposed mechanism may involve alterations in cell membrane permeability resulting from electrostatic interactions between the cationic NanoT particles and the negatively charged fungal cell surface. This permeability suggests a mechanism resembling an anchoring effect that facilitates drug delivery. Since the interaction between NanoT and the fungal surface alone is insufficient to induce significant cellular damage, the presence of AmB is essential to achieving enhanced antifungal activity. Moreover, an indication of AmB superaggregates formation after loaded into NanoT particles was observed by UV–vis data, which can act as a versatile carrier to facilitate a sustained release of parent AmB molecules (as observed in Figure 6b) leading to an augment in the efficacy of antifungal assays using NanoT-AmB. Scheme 2 illustrates a representative model of the potential antifungal mechanism for the NanoT-AmB, highlighting the potential release of AmB from the nanogel and detailing the antifungal pathways associated with the drug’s mechanism of action: pore formation within the cell membrane, the “sponge” model for ergosterol sequestration, and the generation of reactive oxygen species (ROS), ultimately leading to fungal cell destruction.^49,50,65–68^

**Scheme 2:**
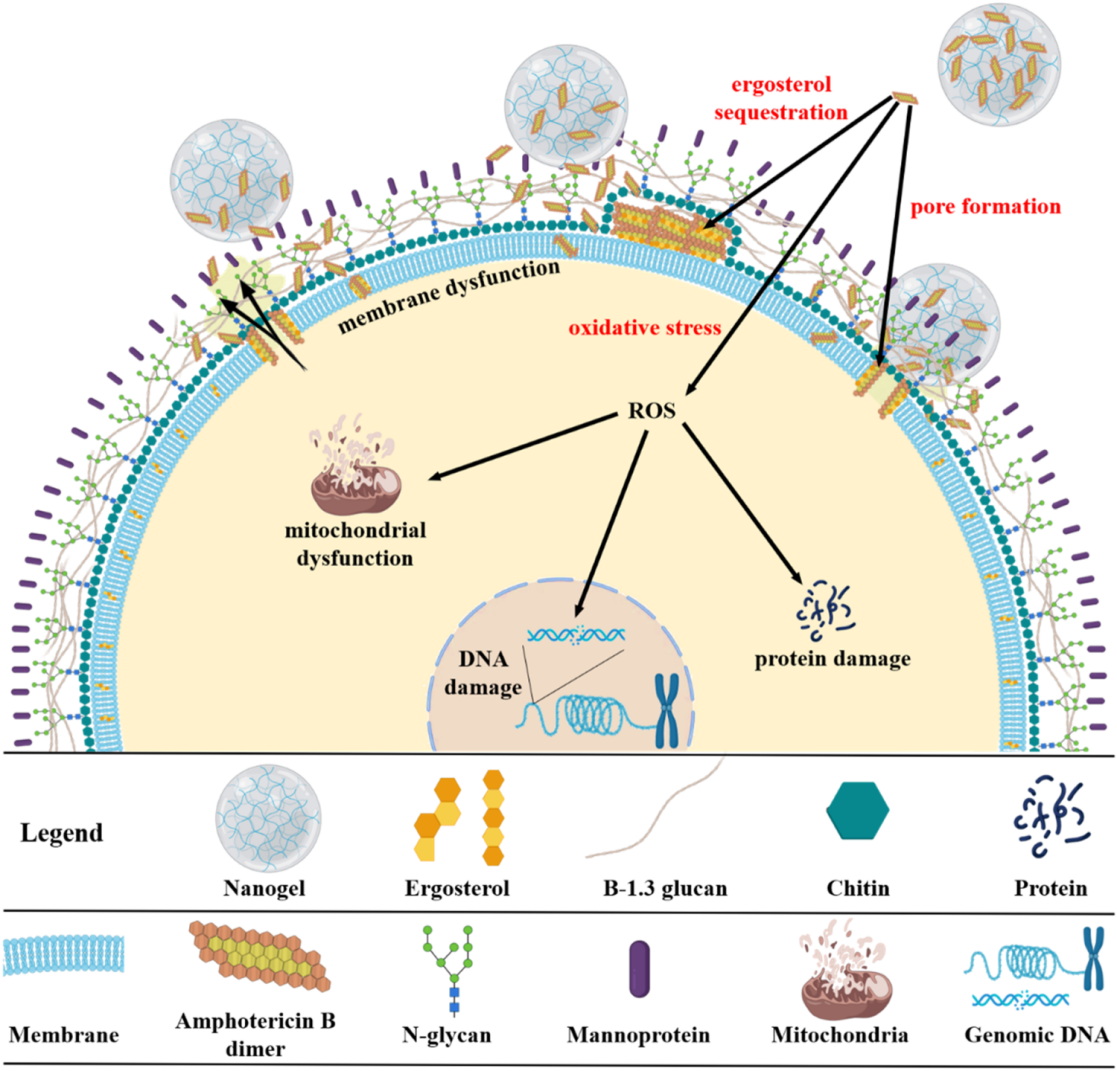
Illustrative Diagram of AmB Release from the Polymeric Network of NanoT403 and Its Fungicidal Action through the Three Proposed Mechanisms of AmB Action: Pore Formation, Ergosterol Sequestration by the Sponge Model, and Oxidative Stress

Currently, it is rare to find formulations of Amphotericin B in the literature and available on the market, though extensive research is directed toward developing innovative formulations to effectively treat fungal infections such as candidiasis and aspergillosis, as well as parasitic diseases like leishmaniasis.^69,70^ Zhou et al.^71^ developed a polymeric micelle system, AmB/MPEG-PCL-*g*-PEI, aiming to use AmB for the local treatment of oral infections caused by *C. albicans*. The micelles demonstrated sustained drug release under simulated oral cavity conditions (pH 6.8) and infectious environments caused by *C. albicans* (pH 5.8). Additionally, the system showed enhanced antifungal activity against the *C. albicans* biofilm, representing a promising strategy for the topical treatment of oral candidiasis. In another study, Inukai et al.^72^ developed polymeric micelles with Dectin-1 based on poly­(ethylene glycol) (PEG) and amino acid block copolymers. These polymeric micelles containing AmB and DEC demonstrated the ability to accumulate in fungi, showing enhanced antifungal activity and highlighting their potential application as a targeted delivery strategy against fungal infections. Amine-epoxide NanoT gels, used as nanocarriers for the AmB drug, present a promising strategy for future applications (feasible via topical administration) aimed at addressing the global challenge of fungal infections, as noted above. Rolón et al.^73^ developed a novel micellar system based on sodium deoxycholate containing amphotericin B (AmB-NaDC) as a new oral dosage form targeting *Trypanosoma cruzi* infections. The study reported that oral administration of AmB-NaDC led to a 75% reduction in parasitemia levels and significantly extended the survival rate to 100% among the test animals. In another study, Ghosh et al.^74^ developed amphotericin B-loaded mannose-modified PLGA nanoparticles aimed at overcoming toxicity and drug resistance in the treatment of visceral leishmaniasis. The antileishmanial efficacy of these AmB-loaded nanoformulations demonstrated superior effectiveness compared to free AmB. The amine-epoxide NanoT gels as a water-based nanocarrier (sustainable system) for AmB drug may show future potential for applications in addressing the significant challenge of parasitic diseases throughout the world as cited above.

The literature has reported that gel nanoparticles derived from epoxide–polyetheramine exhibit high cell viability, supporting their potential for biomedical applications, including the development of controlled-release therapeutics.^15^ As demonstrated by Tang et al.,^15^ *in vitro* studies show that incorporating polyetheramine during nanoparticle synthesis enhances cytocompatibility. Moreover, no cytotoxic effects were observed in mammalian cells exposed to these nanoparticles. Additionally, due to their positively charged surfaces, epoxide–polyetheramine nanoparticles have shown the ability to bind DNA, suggesting their potential use as nonviral vectors in gene therapy through nanotechnological approaches.^15^ Previous studies by our group have demonstrated the good biocompatibility of this class of amine-epoxide-based polymeric gels (as evidenced by *in vitro* XTT assays using a normal human lung fibroblast cell line), and the absence of genotoxic effects (*in vivo* zebrafish assays).^17,18^ As further evidence supporting the bioapplication potential of these nanogels, we assessed the blood compatibility of NanoT. The hemolysis assay confirmed that the nanogels exhibited no hemolytic activity, demonstrating excellent blood compatibility (see Figure S5). These findings further support the safety of NanoT as a potential delivery system for bioactive agents, including those that present poor water solubility such as AmB.

## 4 Conclusions

In this study, we designed a green synthesis of amine-epoxide particles containing AmB molecules as a novel system to improve the therapeutic index of the antifungal. The formation mechanism and nanogel (spherical particles) obtention were investigated by several physicochemical techniques including syncrotron-based ultra-small angle X-ray scattering USAXS. Moreover, computational simulations revealed the noncovalent interaction between nanogel and drug. The encapsulation of AmB exhibited a loading capacity of ∼55%. The incorporation of the antifungal into NanoT gel induces the formation of AmB superaggregates which can facilitate the release of the parent AmB molecules slowly, showing an augment in the efficacy of antifungal assays by 4-fold reduction. Furthermore, the antifungal mechanism of action was found to be related to the release of the AmB drug by the amine-epoxide nanogel and possible electrostatic interactions on the fungal surface. Thus, a nanogel based on amine-epoxide provides a promising approach to develop a water-based and green antifungal carrier for future utilization as controlled-release systems against important fungal pathogens.

## Supplementary Material



## Notes

Details of the USAXS analysis and temporal profile curves evolution as a function of temperature, AmB drug calibration curve, FTIR spectra of monomers and NanoT system, fitting of Amb B release curves by applying different mathematical models, hemolysis assay results, sample details D_h_, PDI, and ζ-potential, encapsulation efficiency calculations for AmB, and mathematical models used to describe AmB drug dissolution curves of the loaded NanoT (PDF)
