# Supplementary material for: Enhanced Antifungal Efficacy through Controlled Delivery of Amphotericin B Loaded in Polyetheramine-Epoxide Nanogels
Source: ACS Polym Au. 2025 Jul 25;5(4):406–19. doi: 10.1021/acspolymersau.5c00037 (PMC12355616; doi:10.1021/acspolymersau.5c00037)
Supplement: Supplementary file 1 [file lg5c00037_si_001.pdf]

ELECTRONIC SUPPLEMENTARY MATERIAL

Enhanced Antifungal Efficacy through Controlled Delivery of Amphotericin B Loaded in Polyetheramine-Epoxide Nanogels

Julia S. Reinaldi[1], Heber E. Andrada[1], Ana F. A. P. Cunha[1], Bruno A. Fico[1], Felipe B. Alves[1], Renato P. Orenha[1], Renato L. T. Parreira[1], Regina H. Pires[1], Fabián Vaca Chávez[2], Carolina E. Tissera[2,3], O. Fernando Silva[2,3], Mariana A. Fernández[2,3], Aline R. Passos[4], Eduardo F. Molina[1]*

[1] Universidade de Franca, Av. Dr. Armando Salles Oliveira 201, Franca, SP, 14404-600, Brazil

[2] Universidad Nacional de Córdoba, Ciudad Universitaria, X5000HUA, Córdoba, Argentina.

[3]Consejo Nacional de Investigaciones Científicas y Técnicas (CONICET), Instituto de Investigaciones en Fisicoquímica de Córdoba (INFIQC), X5000HUA, Córdoba, Argentina.

[4]Brazilian Synchrotron Light Laboratory, Brazilian Centre for Research in Energy and Materials, 13083-1000 Campinas, Brazil

*Corresponding author e-mail: eduardo.molina@unifran.edu.br or molina_ferreira@yahoo.com.br

The USAXS data were fitted using the Guinier-Porod (equation 1) model to determine the size and dimensionality of the scattering structures.[1]

$$I\,(q) = \begin{cases} \dfrac{G}{q^s}\; exp\left[\dfrac{-\,q^2 R_g^2}{3-s}\right] & for\; q\; \leq q_1 \\ \dfrac{D}{q^m}\; for\; q\; \geq q_1 \end{cases}$$

where q is the scatteting vector, $I(q)$ is the scattered intensity, R_g is the gyration radius, m is the Porod exponent, G and D are the Guinier and Porod scale factors, and the s parameter. For globular objects, such as spheres, s = 0; for rods s = 1 and for lamellae s = 2.

Table S1. Parameters obtained from fitting the Guinier-Porod model.

Temperature	R_g	m	s
45 °C	190	3.9	0
50 °C	219	4	0
55 °C	232	3.8	0
60 °C	241	3.9	0

Table S2. Hydrodynamic diameter (D_h), polydispersity index (PDI), and zeta potential (ζ) values for amine-epoxide-based NanoT unloaded and loaded with AmB drug.

Sample	D_h (nm)	PdI	ζ (mV)
NanoT	169 ±23	0.20	+26
NanoT-AmB	270 ±20	0.07	+5.0

Table S3. Mathematical models used to describe AmB drug dissolution curves of the loaded NanoT.

Mathematic model	Equation	Parameters	Reference
Zero order	$Q_t = Q_0 + k_0 t$	Q_t = amount of drug dissolved at time t.<br>Q_0 = initial amount of drug in the solution.<br>K_0 = zero-order release constant.	Varelas et al.[2]
First Order	$LogC_t = LogC_0 - \frac{k_1}{2.303} t$	C_t = remaining amount of drug at time t.<br>C_0 = initial amount of drug in the solution.<br>k_1 = first-order release constant.	Gibaldi et al.[3]
Higuchi	$Q_t = k_H t^{1/2}$	Q_t = amount of drug dissolved at time t.<br>K_H = Higuchi dissolution constant.	Higuchi et al.[4]<br><br>Higuchi et al.[5]
Hixson-Crowell	$W_0 \frac{1}{3} - W_i \frac{1}{3} = K_{HC} t$	W_0= initial amount of drug in the dosage form.<br>W_i= remaining amount in the dosage form at time t.<br>K_{HC}= incorporation constant, which relates surface area and volume.	Costa et al.[6]
Korsmeyer-Peppas	$\frac{M_t}{M_\infty} = k_r t^n$	M_t/M_∞ = The fraction of drug released at time t.<br>K_r = the release constant characteristic of the polymer-drug interactions.<br>n = The diffusion exponent characteristic of the release mechanism.	Korsmeyer et al.[7]<br><br>Peppas et al.[8]

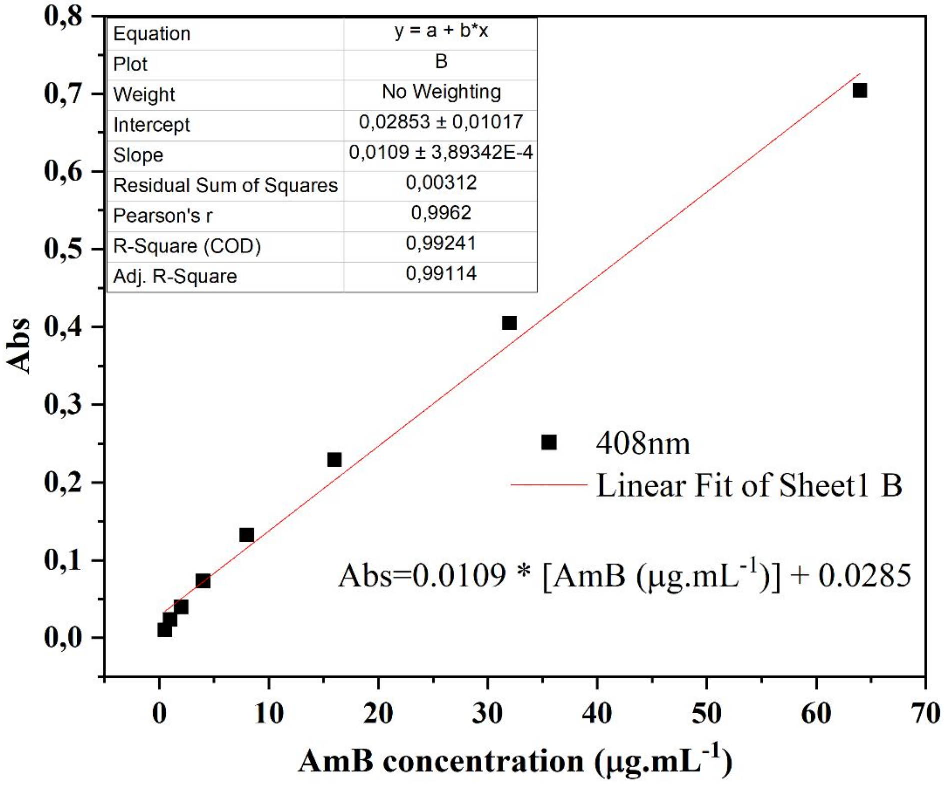

Figure S1. Calibration curve obtained for AmB at 408 nm, for a concentration range from 0 to 64 μg/mL.

Encapsulation Efficiency (%) Calculations for Amphotericin B (AmB)

To determine the encapsulation efficiency (EE%) of AmB, a calibration curve was constructed using standard solutions of AmB in a suitable solvent. The absorbance was measured at 408 nm, which corresponds to the characteristic maximum absorption of AmB. The resulting equation was:

$Abs_{408nm} = 0.0109 \times [AmB] + 0.0285$ ($R^2 > 0.99$, where [AmB] is in μg/mL)

This equation was used to determine the concentration of non-encapsulated AmB in the filtrate after the encapsulation process. From this concentration and the analyzed volume, the total amount of AmB that was not encapsulated was calculated.

The total amount of AmB initially added to the system was 320 μg.

The encapsulation efficiency (EE%) was calculated using the following formula:

$$EE(\%)_{408nm} = \frac{Amount\ of\ encapsulated\ AmB}{Total\ amount\ added}\ x\ 100$$

Where:

- Encapsulated amount = Total amount added – Free amount (in the filtrate), based on the following equation:

$$EE(\%)_{408nm} = \frac{mass_{AmB\ Total} - mass_{AmB\ free}}{mass_{AmB\ Total}}\ x\ 100$$

According to the obtained results, the spectrophotometric analysis of the filtrate yielded an absorbance value that, when applied to the calibration equation, allowed us to calculate the concentration of free AmB. From this value, the amount of non-encapsulated AmB was determined, and by subtracting it from the total added, the amount of encapsulated AmB was obtained.

In this case, the calculations resulted in an encapsulation efficiency of 53%, indicating that approximately 169.6 µg of AmB were effectively encapsulated, while 150.4 µg remained free in the filtrate:

$$EE\% = \frac{169.6\mu g}{320\mu g} \times 100 = 53$$

Considering typical uncertainties in absorbance measurements, pipetting, and sample handling, an estimated relative error of ±5% was assigned to the encapsulation efficiency. Therefore, the encapsulation efficiency can be expressed as:

$$EE\% = (53\ \pm 3\ )\%$$

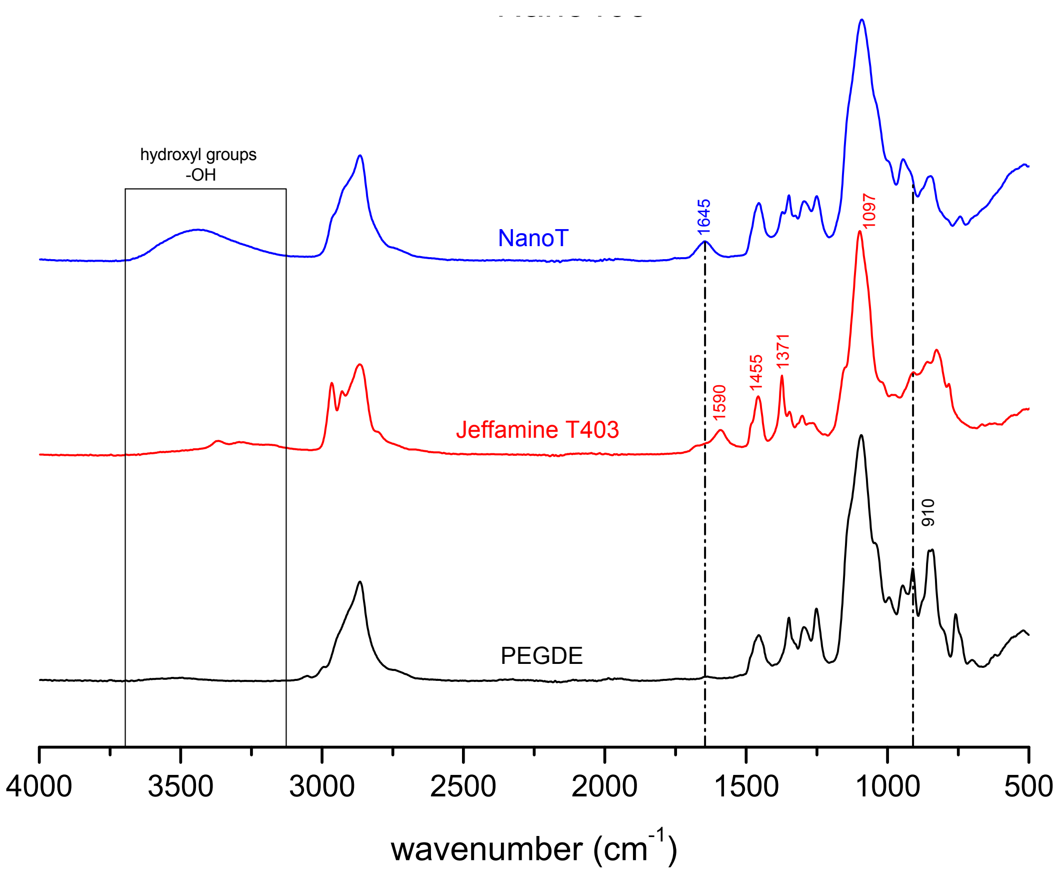

Figure S2. Room-temperature FTIR spectra of PEGDE, Jeffamine T403 and NanoT.

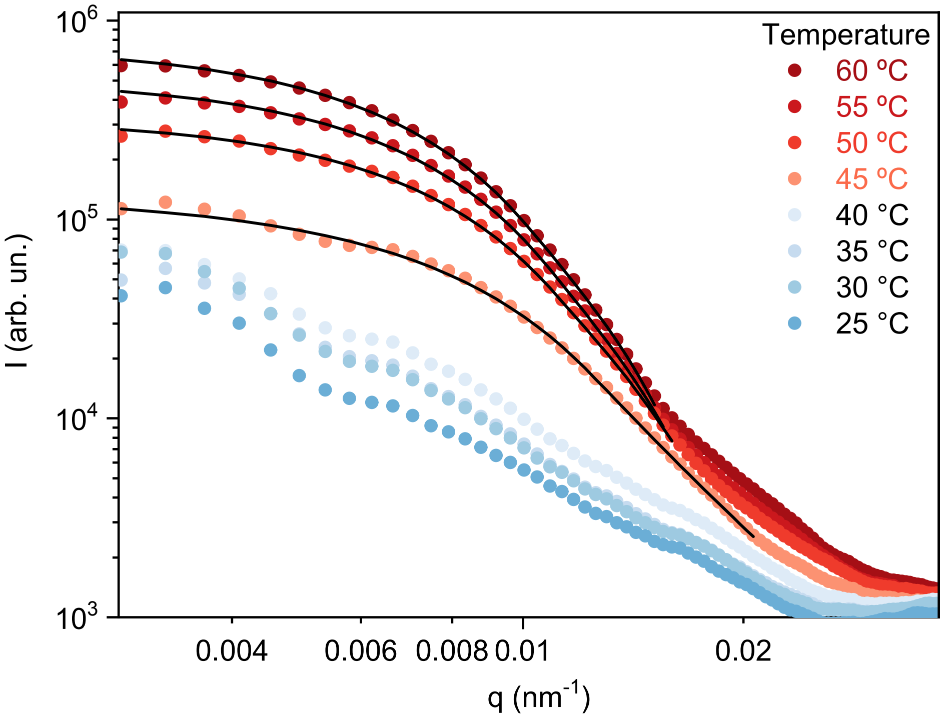

Figure S3. Temporal evolution of the USAXS profile during the synthesis of NanoT particles as a function of temperature.

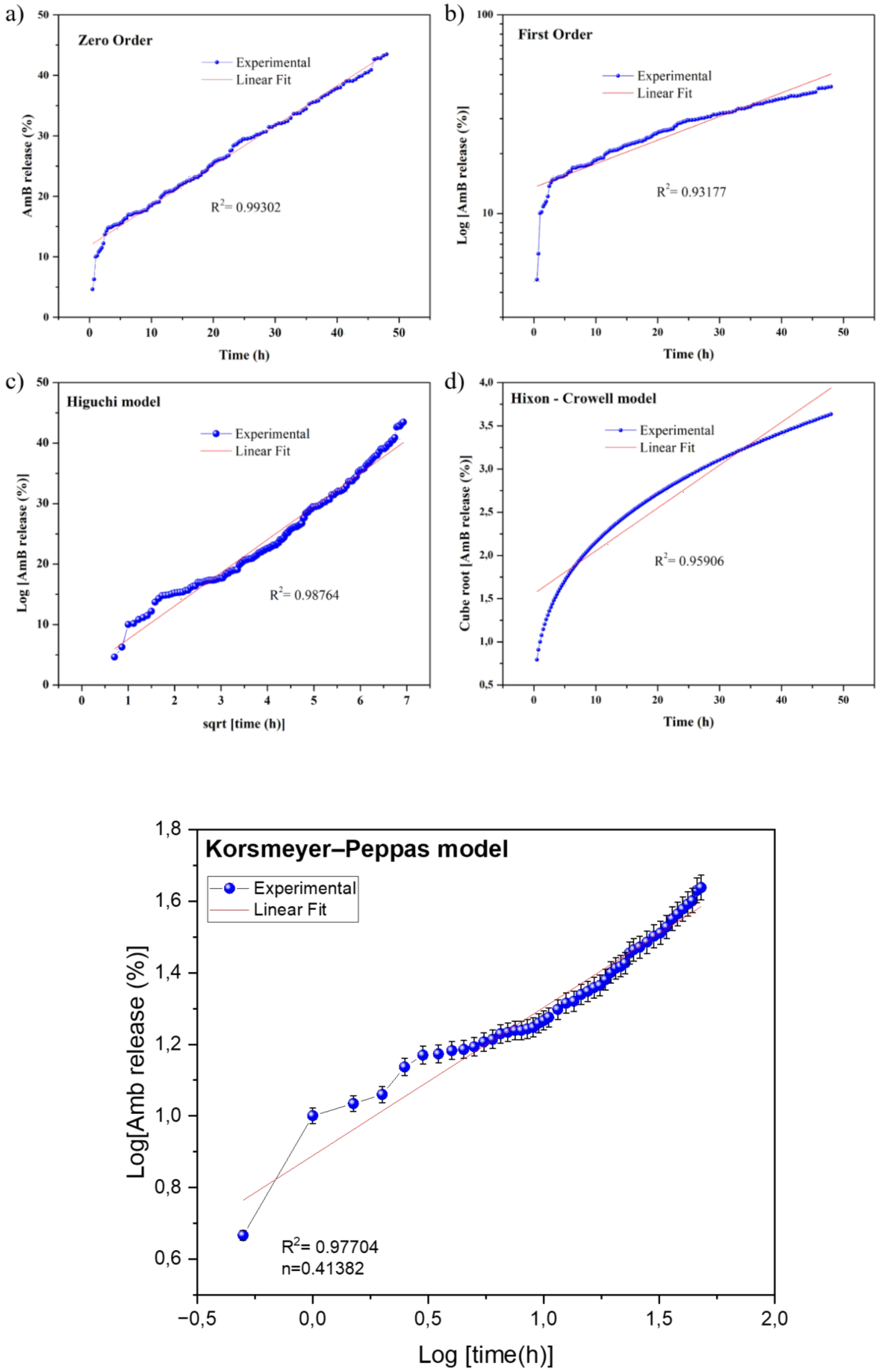

Figure S4. Amphotericin B release curves as a function of time plotted using different mathematical models. Data are represented as mean ± SD from three independent experiments.

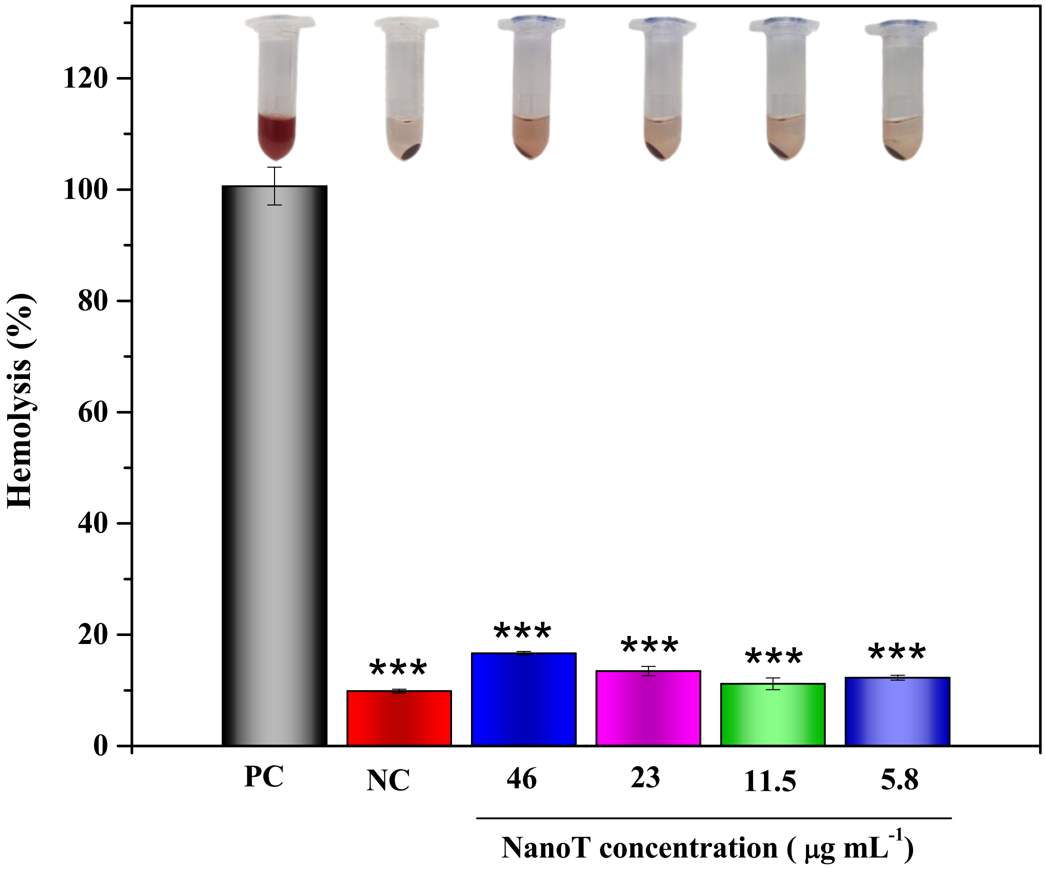

Figure S5. Percentage of hemolysis after 3 hours of incubation with sheep blood and different concentrations of pure NanoT. Data are represented as mean ± SD from three independent experiments. Statistical analysis was performed using two-way ANOVA (*** $p < 0.0001$).

References

(1) Hammouda, B. A new guinier–porod model. *Journal of Applied Crystallography* **2010**, *43* (4), 716–719. DOI: 10.1107/s0021889810015773

(2) Varelas, C. G.; Dixon, D. G.; Steiner, C. A. Zero-order release from biphasic polymer hydrogels. *Journal of Controlled Release* **1995**, *34* (3), 185–192. DOI: 10.1016/0168-3659(94)00085-9

(3) Gibaldi, M.; Feldman, S. Establishment of sink conditions in dissolution rate determinations. Theoretical considerations and application to nondisintegrating dosage forms. *Journal of Pharmaceutical Sciences* **1967**, *56* (10), 1238–1242. DOI: 10.1002/jps.2600561005

(4) Higuchi, T. Rate of release of medicaments from ointment bases containing drugs in suspension. *Journal of Pharmaceutical Sciences* **1961**, *50* (10), 874–875. DOI: 10.1002/jps.2600501018
(5) Higuchi, T. Mechanism of sustained-action medication. Theoretical analysis of rate of release of solid drugs dispersed in solid matrices. *Journal of Pharmaceutical Sciences* **1963**, *52* (12), 1145–1149. DOI: 10.1002/jps.2600521210
(6) Costa, P.; Sousa Lobo, J. M. Modeling and comparison of dissolution profiles. *European Journal of Pharmaceutical Sciences* **2001**, *13* (2), 123–133. DOI: 10.1016/s0928-0987(01)00095-1
(7) Korsmeyer, R. W.; Gurny, R.; Doelker, E.; Buri, P.; Peppas, N. A. Mechanisms of solute release from porous hydrophilic polymers. *International Journal of Pharmaceutics* **1983**, *15* (1), 25–35. DOI: 10.1016/0378-5173(83)90064-9
(8) Peppas, N. A. Analysis of Fickian and Non-Fickian Drug Release from Polymers. *Pharm. Acta Helv.* **1985**, *60* (4), 110–111.
